# Clinical characteristics of pain originating from intra-articular structures of the knee joint in patients with medial knee osteoarthritis

**DOI:** 10.1186/2193-1801-2-628

**Published:** 2013-11-23

**Authors:** Masahiko Ikeuchi, Masashi Izumi, Koji Aso, Natsuki Sugimura, Toshikazu Tani

**Affiliations:** Department of Orthopaedic Surgery, Kochi Medical School, Kochi University, Kohasu, Oko-cho, Nankoku, Kochi, 783-8505 Japan

**Keywords:** Knee, Osteoarthritis, Pain

## Abstract

**Purpose:**

Although disease progression of osteoarthritis has been well documented, pain pathophysiology is largely unknown. This study was designed with two purposes: 1) to characterize patients with knee pain predominantly originating from intra-articular structures and 2) to describe the location and pattern of their pain.

**Materials and methods:**

103 patients with medial knee osteoarthritis underwent an intra-articular injection of local anesthetics (joint block). At least 70% pain relief was defined as positive for the joint block, while less than 50% as negative. Pain characteristics in patients positive for joint block were evaluated in detail using a knee pain map.

**Results:**

Sixty three knees (61%) were positive and 33 knees (32%) were negative. Patients negative for the joint block were significantly higher age, suffered for longer time, and complained more diffuse pain. Although pain at anterior medial area during walk was the most common finding, pain characteristics differed among different knee areas.

**Conclusion:**

The characteristics of joint pain are widely variable even in patients with similar radiological features. Extra-articular sources are not negligible especially in older patients with a long history of diffuse pain. Differences in pain characteristics among knee areas should be taken into account when examining the pain source.

## Introduction

Knee osteoarthritis (OA) is a major public health problem across the world. Population based studies revealed that symptomatic knee OA is present in 20-30% of the elderly population aged >65 years (Mannoni et al. 2003; Andrianakos et al. 2006) and its prevalence is increasing due in part to the aging of the population (Leveille 2004). Clinical symptoms are dominated by chronic knee joint pain, which leads to disability, psychological distress, and impaired quality of life. Recently, patient-oriented outcome measures have been acquiring greater importance in treating knee OA.

Despite significant advances in the understanding of the pathology of knee OA, pain pathology associated with knee OA remains largely unknown (Dieppe & Lohmander 2005).

Recently association between symptom and advanced imaging such as MRI (Hunter et al. 2011) and bone scintigraphy (Kraus et al. 2009) is reported. All innervated tissues inside and around the knee joint are potential pain generators in knee OA. In addition, nerves itself, not only peripheral nerves but also central nervous system, plays a significant role in pain mechanisms in knee OA (Lee et al. 2011). Pain generators may differ widely even among patients with similar radiological features, which can contribute to variability in treatment response. It was hypothesized that there would be considerable variation of pain characteristics among patients with similar radiological features. Herein, we examined the main pain sources of medial compartment OA, which is the most common form of knee OA, utilizing a diagnostic joint block, in particular reference to intra-articular tissues. Our study was designed with two purposes: 1) to characterize patients with knee pain predominantly originating from intra-articular structures and 2) to describe the location and pattern of their pain.

## Materials and methods

A prospective study on patients with knee pain associated with medial OA presenting to a rural medical college hospital was conducted over a 1 year interval (August 2008 – July 2009). Inclusion criteria were American College of Rheumatism clinical criteria (Altman et al. 1986), 100-mm pain visual analogue scale (VAS) greater than 30 mm, radiological OA predominantly localized to medial tibiofemoral joint with grade≧2 according to the Kellgren-Lawrence grading system (0 = none, 1 = doubtful, 2 = minimal, 3 = moderate and 4 = severe) (Kellgren & Lawrence 1957). The pain VAS was used to assess the pain intensity in the knee joint. Patients were asked to rate their knee pain for the last week by placing a mark on a 100 mm line with two-endpoints representing “no pain (0)” and “worst pain imaginable (100)”. The distance along the line from the “no pain” marker was then measured with a ruler giving a pain score out of 100. Pain VAS greater than 30 mm means moderate to severe knee pain. Exclusion criteria were radiological OA involving lateral tibiofemoral joint or patellofemoral joint with grade≧2, inability to walk 200 m within 5 minutes, mental handicap or psychiatric condition precluding adequate communication, coagulation disturbances, allergies to local anesthetics, previous knee surgeries, and pain originating from hip and spine pathology. Physical examination on hip joints and spine were routinely performed during initial pain assessment. In particular, radiating pain and referred pain associated with motion of hip and spine were examined and excluded in this study. Japanese Orthopaedic Association (JOA) knee score (Wakabayashi et al. 2002), ranging from 0 (worst) to 100 (best), was used to evaluate the clinical state. It consists of four domains: pain on walking (0–30 points), pain on ascending or descending stairs (0–25 points), range of motion (0–35 points), and joint effusion (0–10 points) (Okuda et al. 2012).

**Figure 1:**
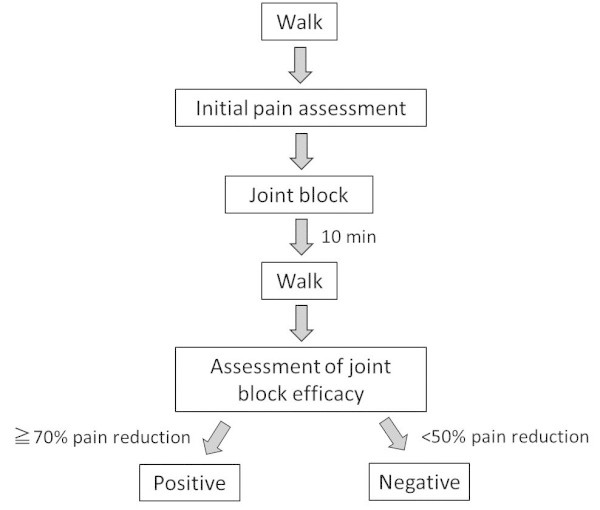
**Experimental protocol.**

**Figure 2:**
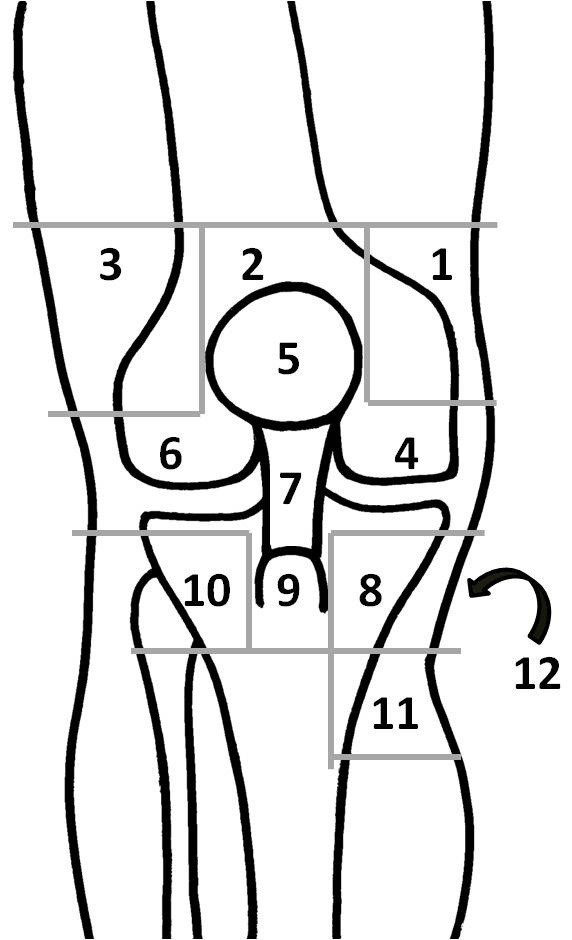
**Knee pain map.** Representative anatomical structures within each numbered area are described in main text in details.

The experimental protocol is summarized in Figure 1. Before the initial assessment of knee pain, patients were instructed to walk 200 m in a hallway at a comfortable speed within 5 minutes. Immediately after the walk, patients were asked to draw a line with pen on their own knees to show the painful area. Then an examiner copied the painful area on a knee pain map developed by (Hill et al. 2003). There are 12 areas with grid lines in this knee pain map (representative anatomical structures within the area); 1 anterior supero-medial (vastus medialis), 2 anterior superior (quadriceps tendon), 3 anterior supero-lateral (iliotibial band and vastus lateralis), 4 anterior medial (medial joint line and medial collateral ligament), 5 patella, 6 anterior lateral (lateral joint line and lateral collateral ligament), 7 patella tendon, 8 pes anserinus, 9 tibial tuberosity, 10 anterior infero-lateral (fibula head and tibialis anterior), 11 anterior infero-medial (tibial insertion of the superficial medial collateral ligament), 12 popliteal (Figure 2). The summation of painful areas was recorded as total painful area. Knee pain during passive extension and flexion was also assessed using the same knee pain map. Examiners (MI and MI) held the patient’s leg and gently moved the knee joint to the end of range without over-pressure. Finally, tender points in each area were assessed manually with the patient lying down with knees straight. Examiners applied pressure using the thumb pad to the approximate center of each area for 3 seconds. The pressure force was equivalent to 4 kg, which was sufficient to blanch the nail bed. For a tender point to be considered ‘positive’, the patient must state that the palpation was painful. If the patient complained of pain in both knees, the more severely affected side was evaluated.

Standard superolateral approach (Zuber 2002) was used for intra-articular injection. A 20G needle was inserted from superolateral side in a sterile manner, and introduced into the suprapatellar pouch, which is a large continuation of the synovial membrane of the knee joint proximal to the trochlea (Dragoo & Abnousi 2008). Following joint fluid aspiration, 5 ml of 1% lidocaine were injected into the knee joint, and then 2.5 ml hyaluronic acid (Artz, Kaken seiyaku, Japan) were injected using a same needle. Because previous *in vitro* studies have shown that local anesthetics are chondrotoxic to human articular cartilage (Grishko et al. 2010; Piper et al. 2011), hyaluronic acid was added in expectation of protecting articular cartilage (Hester et al. 2012; Liu et al. 2012). The knee joint was passively moved from extension to flexion 5 times. Ten minutes after the injection, the patients were instructed to walk 200 m at a comfortable speed, and then pain assessment was performed as the same as initial assessment. According to previous reports using stringent pain relief criteria (Lovely & Rastogi 1997; Broadhurst & Bond 1998; Yeom et al. 2008), a minimum reduction of 70% in the VAS rating during walk was required to be considered a positive response. A negative response was defined as reduction less than 50%, which is the most commonly used cutoff in the current literature regarding diagnostic blocks (Schwarzer et al. 1994; Liliang et al. 2009; Rupert et al. 2009; Choi et al. 2011). The changes in the area of pain following injection were not evaluated because they were unremarkable. All procedures and assessments were performed by two experienced knee surgeons (MI and MI).

Ethics committee approval from our institution was obtained prior to the study. Before study inclusion, each patient was informed of the objectives and risks of the study and gave his or her consent. The study was conducted in compliance with the Declaration of Helsinki.

In this study, we expediently defined “intra-articular tissues” as joint structures which can be infiltrated with 5 ml of local anaesthetics with 2.5 ml hyaluronic acid, including synovium, cruciate ligaments, exposed subchondral bone and cartilage.

Statistical analyses were performed with the SPSS statistical package for Windows. As this was an observational study, we did not conduct any power analysis for sample size estimation. Mann–Whitney test was used to compare the background characteristics except Kellgren-Lawrence grade 4 (Chi-square test) between joint block-positive and negative patients. *P* <0.05 was regarded as statistically significant.

## Results

One hundred-three patients with medial knee OA were included in this study. There were 85 women and 18 med with a mean age of 72 years (52 to 85). All patients underwent the diagnostic joint block and main sources of knee pain were examined. Sixty-three patients (61%) had positive response to the joint block, while thirty-three patients (32%) were regarded as negative. Seven patients who had a 50-70% pain reduction after joint block were excluded from the analysis. Table 1 shows the result of comparison of baseline characteristics between joint block-positive and negative patients. Patients with negative response were significantly higher age (*p* = 0.016), suffered knee pain for longer time (*p* = 0.024), and complained more diffuse pain (*p* = 0.003). Neither radiological severity nor clinical score were associated with block effects (Table 1).

**Table 1:** **Comparison of baseline characteristics between joint block-positive and negative patients**

	Response to joint block		***P***value
	Positive	Negative	
Age (yrs)	70 (10)	76 (6)	0.016*
Disease duration (mo)	72 [12, 180]	120 [50, 240]	0.024*
JOA knee score (pts)	63 (14)	58 (15)	0.090
Pain BAS during walk (mm)	64 (19)	62 (15)	0.653
Kellgren-lawrence grade 4 (%)†	48	48	0.995
Knee range of motion (deg)	122 (22)	125 (17)	0.708
Total painful area	2 [1, 4]	4 [3, 7]	0.003**

Values are mean (standard deviation) and median [inter-quartile range]. Mann–Whitney test was used except † Chi-square test. *p < 0.05 and **p < 0.01.

**Figure 3:**
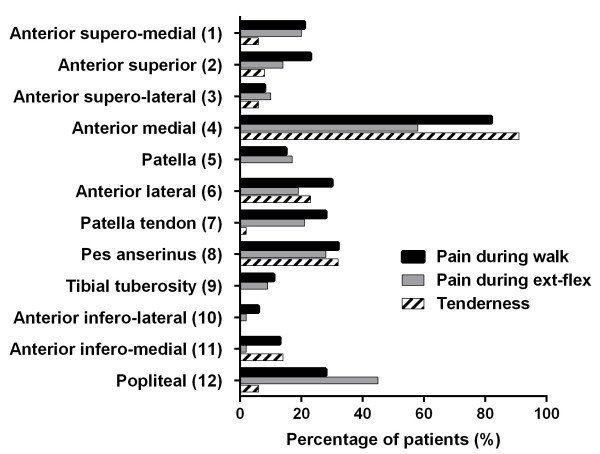
**Percentage of patients with pain and tenderness in each area.** The number in parentheses is equal to the number in the knee pain map (Figure 2). ex: extension-flexion.

Among patients in the positive group, the highest prevalence (82%) of pain during walk was observed at anterior medial area (area 4), followed by pes anserinus area (area 8) (32%) and anterior lateral area (area 6) (30%) (Figure 3). In addition, pain during walk was sometimes referred to thigh (3 patients) and lower leg (10 patients). Although the highest prevalence (58%) of pain during extension-flexion of the knee was also observed at anterior medial area (area 4), the next highest prevalence (45%) was popliteal area (area12) (Figure 3). Specifically, popliteal pain was elicited during flexion in 18 patients, extension in 7 patients and both extension and flexion in 3 patients. Tender points were predominantly localized near the joint line (area 4, 6) and pes anserinus (area 8) (Figure 3).

## Discussion

Our results showed that intra-articular tissues were significant source of pain for 61% patients with medial knee OA. However, older patients with a long history of diffuse pain tended to have negative response to the joint block, indicating the contribution of intra-articular tissues to knee pain was relatively small. One possible explanation for this result is subsequent extra-articular pathology such as decreased elasticity of the soft tissues (Fishkin et al. 2002), neo-innervation of the degenerated tissues (Lian et al. 2006), and central sensitization (Finan et al. 2013). In order to achieve effective pain management for those patients, not only intra- but also extra-articular pathology should be targeted. Pain management targeting extra-articular pathology include physical therapy for muscle and soft tissues, drugs such as muscle relaxant and centrally acting analgesics, and cognitive behavioral therapy.

Pain in knee OA involves the peripheral and central nervous system (Lee et al. 2011). Inflammatory mediators inside the joint sensitize nociceptors innervating synovium, leading to local areas of enhanced pain sensitivity. Eventually, growth of new nociceptors and activation of nociceptors in the exposed subchondral bone augment the peripheral input to the central nervous system. Central pain resulted from central sensitization and reduced central inhibition leads to prolonged and enhanced pain (Sluka et al. 2012). In this study, pain originating from intra-articular structures in medial knee OA was not always localized at synovial cavity of the joint, let alone medial side. For instance, although there is no synovial cavity in area 8 (around pes anserinus), pain was relieved by joint block in the positive group. If the local insults caused pain in area 8, joint block was theoretically not effective. Therefore, we think that the pain mechanism of diffuse pain is at least partly due to alterations in central pain processing.

Pain characteristics differed among different knee areas. For instance, pain at anterior medial area (area 4) was the most common finding in patients with medial knee OA. This pain was observed during walk rather than during extension-flexion, which might reflect increased load at medial compartment in genu varum. On the contrary, popliteal pain (area 12) was observed during extension-flexion of the knee rather than during walk, which might reflect impingement of torn meniscus and capsular contracture. In addition, pain at anterior medial area was usually accompanied by tenderness, while popliteal pain was not. It is possible that the presence of tenderness only reflects the superficial location of painful structures. However, knowing the pain characteristics at each knee area is helpful in evaluating pain in knee OA during daily practice.

Several authors used diagnostic blocks with a criterion of 50% pain relief for knee joint (Choi et al. 2011), sacroiliac joint (Rupert et al. 2009), lumbar facet joint (Schwarzer et al. 1994), and shoulder joint (Liliang et al. 2009). In this study, a more stringent criterion, 70% pain relief according to (Broadhurst & Bond 1998), was used. A response of 70% pain relief could be inferred to indicate that the intra-articular tissues were not sole but significant source of pain. Although 70% criteria were to decrease the false-positive rate, it might have resulted in increased false-negative cases vice versa. It seemed more problematic to include the false-positive cases because the main purpose of this study was not to clarify the prevalence of pain originated from intra-articular tissues but to describe its characteristics.

The strength of this study is detailed pain assessment using joint block. However, there are several limitations in our study. Firstly, a placebo response might have been involved because of our single uncontrolled block. For an ideal prevalence study, a placebo controlled or comparative local anaesthetic study will be necessary. Secondly, intra-articular injection was performed blindly. Although the superolateral approach is reported as the most accurate approach for intra-articular knee injection (Hermans et al. 2011), ultrasound guidance (Berkoff et al. 2012) might have resulted in the better accuracy of injection. Thirdly, spread of local anaesthetics could be individually variable. Future clinical trials using a double-injection paradigm for patient selection and a control group are needed to further clarify the pain referral patterns observed in knee OA. Lastly, the problems with the study design include observational study with relatively small sample size. Despite these limitations, we believe that this study provides clinically important information on pain characteristics associated with medial compartment knee OA.

In conclusion, our results suggest that the characteristics of joint pain are widely variable even in patients with similar radiological features. Although the main source of pain in medial osteoarthritis knees is intra-articular structures, extra-articular sources are not negligible especially in older patients with a long history of diffuse pain. Differences in pain characteristics among knee areas should be taken into account when examining the pain source of knee osteoarthritis.
